# Impact of surgical risk factors for non-union on lumbar spinal fusion outcomes using cellular bone allograft at 24-months follow-up

**DOI:** 10.1186/s12891-024-07456-4

**Published:** 2024-05-03

**Authors:** Anthony Russo, Daniel K Park, Todd Lansford, Pierce Nunley, Timothy A Peppers, Joshua J Wind, Hamid Hassanzadeh, Joseph Sembrano, Jung Yoo, Jonathan Sales

**Affiliations:** 1 Yellowstone Orthopedic and Spine Institute, Billings Clinic Bozeman, 3905 Wellness Way, 4534 Apt A Perry Street, Bozeman, MT USA; 2 Beaumont Hospital, 3601 W 13 Mile Rd, Royal Oak, MI USA; 3 South Carolina Sports Medicine, 9100 Medcom, N Charleston, SC USA; 4 Spine Institute of Lousiana, 1500 Line Ave, Shreveport, LA USA; 5 Scripps Memorial Hospital Encinitas, 354 Santa Fe Drive, Encinitas, CA USA; 6 Sibley Memorial Hospital, 5255 Loughboro Rd. NW, Washington, DC, USA; 7 John Hopkins Medicine, 6420 Rockledge Dr, Bethesda, MD USA; 8 University of Minnesota, 909 Fulton St SE, Minneapolis, MN USA; 9 Oregon Health and Science University Hospital, 3303 S Bond Ave, Portland, OR USA; 10 Summit Spine, 9155 SW Barnes Rd. 210, Portland, OR USA

**Keywords:** Lumbar fusion, Arthrodesis, Cellular allograft, Trinity elite, Operative risk factors

## Abstract

**Background:**

The current report investigates fusion rates and patient-reported outcomes following lumbar spinal surgery using cellular bone allograft (CBA) in patients with risk factors for non-union.

**Methods:**

A prospective, open label study was conducted in subjects undergoing lumbar spinal fusion with CBA (NCT 02969616) to assess fusion success rates and patient-reported outcomes in subjects with risk factors for non-union. Subjects were categorized into low-risk (≤ 1 risk factors) and high-risk (> 1 risk factors) groups. Radiographic fusion status was evaluated by an independent review of dynamic radiographs and CT scans. Patient-reported outcome measures included quality of life (EQ-5D), Oswestry Disability Index (ODI) and Visual Analog Scales (VAS) for back and leg pain. Adverse event reporting was conducted throughout 24-months of follow-up.

**Results:**

A total of 274 subjects were enrolled: 140 subjects (51.1%) were categorized into the high-risk group (> 1 risk factor) and 134 subjects (48.9%) into the low-risk group (≤ 1 risk factors). The overall mean age at screening was 58.8 years (SD 12.5) with a higher distribution of females (63.1%) than males (36.9%). No statistical difference in fusion rates were observed between the low-risk (90.0%) and high-risk (93.9%) groups (*p > *0.05). A statistically significant improvement in patient-reported outcomes (EQ-5D, ODI and VAS) was observed at all time points (*p < *0.05) in both low and high-risk groups. The low-risk group showed enhanced improvement at multiple timepoints in EQ-5D, ODI, VAS-Back pain and VAS-Leg pain scores compared to the high-risk group (*p < *0.05). The number of AEs were similar among risk groups.

**Conclusions:**

This study demonstrates high fusion rates following lumbar spinal surgery using CBA, regardless of associated risk factors. Patient reported outcomes and fusion rates were not adversely affected by risk factor profiles.

**Trial registration:**

NCT 02969616 (21/11/2016).

## Background

The occurrence of lumbar spinal surgery has increased steadily due to the aging population, advancing diagnostics for degenerative spine disease, improvements in surgical techniques, and the number of qualified surgeons [1, 2]. However, serious complications including non-union, the development of new pain or recurrent symptoms, infection, and further degeneration can contribute to worsening of patient outcomes and the need for revision surgery [3]. These complications can impact a patient's recovery and increase post-operative morbidity. While surgical procedures have advanced, the revision rate remains consistent with a 10-year reoperation rate of approximately 20% [4]. Certain risk factors are known to contribute to lower fusion rates and poorer patient prognosis (e.g., smoking, diabetes, osteoporosis, advanced age, multi-level surgery). Identification and quantification of risk factors contributing to complications following surgery are important for clinical treatment plans and successful patient outcomes. Considering the high prevalence and long-term impact of degenerative lumbar spine disease, exploration into surgical strategies that optimize patient success despite risk factor profiles are imperative.

Choice of bone graft material is a modifiable parameter that may impact the success of lumbar spinal surgery. Autologous bone graft (autograft) has been traditionally considered the gold standard in bone grafting material. Autograft has well documented limitations, including donor site morbidity, graft volume availability, increased operative time, blood loss, post-operative pain, risk of infection, and neurologic injury [5–8]. Complications are observed in up to 38% of autograft procedures [9–11]. Complications associated with autograft harvest, as well as the potential limited quantity and quality of autograft, have led to the evaluation of alternative bone graft substitutes and/or replacements. Cellular bone allografts (CBA) are derived from deceased human donors and are carefully processed and cryopreserved to maintain native, viable osteogenic cells in their cancellous bone components and endogenous bone morphogenetic proteins (BMPs) in their demineralized cortical bone components. CBAs have demonstrated fusion results similar to autologous bone, with a favorable safety profile [6, 8–16]. The efficacy of CBA in patients with risk factors for non-union and complications has not been widely studied. The current report aimed to further investigate spinal fusion rates following surgery which employed CBA as the primary (> 50% by volume) bone graft substance in patients considered low-risk (≤ 1 risk factors) compared to those with high-risk (> 1 risk factors) for non-union and complications. Data for this report was collected from a 24-month, prospective, open-label, multi-center clinical study.

## Methods

### Study participants

As described previously in a 12-month analysis of the trial outcomes [17], subjects were eligible for enrollment if they were over 18 years of age, had failed at least 6 months of conservative care, who planned to undergo posterolateral fusion (1–4 levels) or interbody fusion (1–2 levels), and met the pre-defined inclusion/exclusion criteria. Subjects were excluded if they had prior lumbar spine fusion surgery at a level currently scheduled for surgery, were currently undergoing treatment for malignancy, or had undergone treatment for malignancy within the past 5 years (benign skin cancer permitted), had an active local or systemic infection or were undergoing adjunctive treatment for local or systemic infection. Subjects were enrolled only following informed consent. This study was conducted in compliance with the protocol, Good Clinical Practice guidelines, and all other applicable regulatory requirements and performed in adherence to the guidelines of the Declaration of Helsinki. The study was registered as NCT02969616 (21/11/2016).

### Study design

The current study employed a prospective, post-market, multi-center, open label study design to evaluate efficacy and safety outcomes through 24-months stratified by specified risk factors. Risk factors for non-union included body mass index (BMI), smoking, age, diabetes, osteoporosis, multi-level surgery and the existence of multiple risk factors. High-risk BMI was defined as ≥ 30 kg/m^2^ (BMI of 30 kg/m^2^ or higher is considered obese) [18]. Smoking was defined as subjects who currently were using nicotine (i.e., cigarettes, chewing tobacco, cigars, e-cigarettes, etc.). High-risk age was defined as those over the age of 65 (age legally defined as a senior citizen in the U.S.). Occurrence of diabetes and osteoporosis was confirmed by subject reporting and by medical records, if available. Multi-level surgery was confirmed by subject reporting and the principal investigator (PI)/ surgeon. The occurrence or more than one of these risk factors was required for multi-risk factor designation. Based on these risk factors, subjects were categorized into the high-risk (> 1 risk factor) or low-risk (≤ 1 risk factors) groups. Subjects were prospectively enrolled at nine clinical sites across the United States. A total of 274 subjects were enrolled and included in this analysis.

The surgical approach, technique, and placement/location of the bone graft were determined at the discretion of the treating surgeon. Subjects received CBA using Trinity ELITE matrix (Trinity Elite, MTF Biologics, Edison NJ). Trinity ELITE was used as the primary (> 50% by volume) bone graft substance, with augmentation of up to 50% of locally harvested autograft and/or cancellous allograft chips. No additional bone graft substitutes were allowed.

Radiographic fusion was assessed by an independent review (TELOS Partners, Warsaw, IN, and MMI, Houston, TX). Successful fusion was defined using multi-factor assessment including 1) lack of angular and translational motion (< 3 deg and < 3 mm, respectively) on Quantitative Motion Analysis (QMA) and 2) the presence of bridging bone across the adjacent endplates for interbody fusion or across the transverse processes for posterolateral fusion on thin-cut CT scans. Both fusion criteria had to be met for the subject to be considered a fusion success. Subjects undergoing multi-level procedures had to demonstrate fusion success at all treated levels to be considered a fusion success. Dynamic x-rays (flexion/extension) for QMA were obtained at 3 months, 6 months, 12 months, and 24 months postoperatively, while CT scans were obtained at 12 months and 24 months.

Patient-reported clinical outcomes included Quality of Life (EQ-5D), Oswestry Disability Index (ODI) and Visual Analog Scales (VAS) for back and leg pain. Clinical outcomes were obtained at baseline, 6 weeks, 3 months, 6 months, 12 months, and 24 months post-operatively. Adverse events (AE) were recorded from surgery through 24 months post-operative for each subject, including the event’s relatedness, severity, and outcome.

### Statistical analysis

Data were analyzed with SAS Version 9.4. Counts and percentages are reported for categorical baseline variables. Mean, standard deviation (SD) and range are reported for continuous variables. Pre-operative and post-operative patient-reported outcomes were compared with a Paired Samples T-test. Alpha was set at 0.05 and a p-value < 0.05 was considered significant. Probability for continuous variables (mean differences) was calculated using Mann Whitney U Test as nonparametric alternative to t-test. Probability for categorical variables was calculated using Fisher’s exact test for cell numbers below 5. All available data at each timepoint was included for each analysis.

## Results

### Participants

A total of 274 subjects were enrolled into the study that underwent a surgical fusion procedure. Throughout the course of the study, 17 subjects withdrew their informed consent, 4 subjects were withdrawn from the study by the PI, 2 subjects did not complete follow-up due to an AE, 2 subjects died, and 29 subjects were lost to follow-up. The proportion of enrolled subjects that completed a 12-month follow-up visit, and 24-month follow-up visit was 86.9% and 75.2%, respectively. The proportion of subjects that were lost to follow-up or missed the 12-month and 24-month visit was 4.7% and 15.7%, respectively. The proportion of available subjects that completed the 12 and 24-month follow-up visit was 91.5% and 82%, respectively.

The overall mean age at screening was 58.8 years (SD 12.5) with a higher distribution of females (63.1%) than males (36.9%). The majority of subjects were of Caucasian or white race (86.5%) and not of Hispanic or Latino ethnicity (96.4%). Subjects had an average height of 168.18 cm (SD 10.2) and weight of 86.8 (SD 19.8) with a BMI of 30.7 (SD 6.4) kg/m^2^. A total of 51.1% (*n* = 140) of subjects were categorized into the high-risk group (> 1 risk factor) and 48.9% (*n* = 34) of subjects into the low-risk group (≤ 1 risk factors). Demographics including work status, age, weight and BMI were significantly different between low and high-risk groups (p ≤ 0.001). Compared to low-risk subjects, high-risk subjects were more likely to work part-time or not be working, be older, have an increased weight and BMI index (Table 1).

**Table 1:** Subject demographics by number of risk factors. Demographics were stratified at baseline by subjects in low-risk (≤ 1 risk factor) and high-risk (> 1 risk factors) groups. Work status, age, weight, and BMI were significantly different between risk factor groups

Variable	> 1 Risk Factors	≤ 1 Risk Factor	Overall	*p* – value
Total	140 (51.1)	134 (48.9)	274 (100.0)	-
Sex [n (%)]				
Female	88 (62.9)	85 (63.4)	173 (63.1)	0.92
Male	52 (37.1)	49 (36.6)	101 (36.9)	
Ethnicity [n (%)]				
Hispanic or Latino	4 (2.9)	5 (3.7)	9 (3.3)	0.87
Not Hispanic or Latino	135 (96.4)	129 (96.3)	264 (96.4)	
Unknown	1 (0.7)	0 (0.0)	1 (0.4)	
Race [n (%)]				
Black or Africa American	12 (8.6)	15 (11.2)	27 (9.9)	0.79
Other	5 (3.6)	5 (3.7)	10 (3.7)	
Caucasian or White	123 (87.9)	114 (85.1)	237 (86.5)	
Work Status [n (%)]				
Full Time	38 (27.1)	65 (48.5)	103 (37.6)	0.001
Part Time	13 (9.3)	7 (5.2)	20 (7.3)	
Not Working	89 (63.6)	62 (46.3)	151 (55.1)	
Age (years)				
Mean (SD)	62.0 (11.8)	55.4 (12.3)	58.8 (12.5)	< 0.0001
Min–Max	23–84	19–81	19–84	
Height (cm)				
Mean (SD)	167.14 (10.7)	169.3 (9.5)	168.2 (10.2)	0.06
Min–Max	132.1–195.6	144.8–200.7	132.1–200.7	
Weight (kg)				
Mean (SD)	91.2 (19.0)	82.3 (19.7)	86.8 (19.8)	< 0.0001
Min–Max	41.7–131.5	45.4–163.3	41.7–163.3	
BMI (kg/m2)				
Mean (SD)	32.6 (6.1)	28.6 (6.1)	30.7 (6.4)	< 0.0001
Min–Max	18.0–51.4	18.8–45.2	18.0–51.4	

All risk factors (BMI, smoking, age, diabetes, osteoporosis, multi-level surgery, and multiple risk factors) were significantly different between low-risk and high-risk groups. Subjects in the high-risk group had a higher age and BMI (BMI ≥ 30), were more likely to be smokers, diabetic, have osteoporosis, and were undergoing multi-level surgery (*p* ≤ 0.0003) (Table 2).

**Table 2:** Risk factor by distribution. All risk factor comparisons were significantly different between low risk (≤ 1 risk factor) and high-risk (> 1 risk factors) groups. Subjects with multiple risk factors had a higher age and BMI (BMI ≥ 30), were more likely to be smokers, diabetic, have osteoporosis, and were undergoing multi-level surgery

Variable	> 1 Risk Factors	≤ 1 Risk Factor	Overall	*p* – value
Total	140 (51.1)	134 (48.9)	274 (100.0)	-
BMI Risk Factor				
BMI ≥ 30	108 (77.1)	32 (22.9)	150 (54.7)	< 0.0001
BMI < 30	42 (31.3)	92 (68.7)	124 (45.3)	
Smoking Risk Factor				
Smoker	37 (26.4)	13 (9.7)	50 (18.3)	0.0003
Non-Smoker	103 (73.6)	121 (90.3)	224 (81.8)	
Age Risk Factor				
Age 65 +	75 (53.6)	26 (19.4)	101 (36.9)	< 0.0001
Age < 65	65 (46.4)	108 (80.6)	173 (63.1)	
Diabetes Risk Factor				
Diabetes	47 (33.57)	5 (3.7)	52 (19.0)	< 0.0001
No Diabetes	93 (66.43)	129 (96.3)	222 (81.0)	
Osteoporosis Risk Factor				
Osteoporosis	21 (15.0)	0 (0.0)	21 (7.7)	< 0.0001
No Osteoporosis	119 (85.0)	134 (100.0)	253 (92.3)	
Multiple Levels				
Multiple Levels	61 (43.6)	11 (8.2)	72 (26.3)	< 0.0001
One Level	79 (56.4)	123 (91.8)	202 (73.7)	

### Clinical outcomes

#### Fusion success

No statistical difference in fusion rates were observed between the low-risk (90.0%) and high-risk (93.9%) groups (*p > *0.05) at 24 months. When stratified by risk factor, no difference in fusion success was observed between low or high-risk groups with or without individual risk factors (Table 3).

**Table 3:** Fusion success by risk factor. No statistically significant difference was observed between successful and non-successful fusion by independent risk factor within either risk group

Variable	Overall n	Successful Fusion n	Non-Fusion n	*p* – value
Total	199	183	16	-
BMI Risk Factor				
BMI ≥ 30	101	96	5	0.12
BMI < 30	98	87	11	
Smoking Risk Factor				
Smoker	34	32	2	1.00
Non-Smoker	165	151	14	
Age Risk Factor				
Age 65 +	80	73	7	0.76
Age < 65	119	110	9	
Diabetes Risk Factor				
Diabetes	39	35	4	0.52
No Diabetes	160	148	12	
Osteoporosis Risk Factor				
Osteoporosis	20	18	2	0.67
No Osteoporosis	179	165	14	
Multiple Levels				
Multiple Levels	55	51	4	1.00
One Level	144	132	12	
Multiple Risk Factors				
Multiple Risk Factors	99	93	6	0.27
One Risk Factor	75	69	6	
No Risk Factors	25	21	4	
Multiple Risk Factors				
High Risk Factors (> 1)	99	93	6	0.44
Low Risk Factor (≤ 1)	100	90	10	

#### Quality of life (EQ-5D)

**Fig. 1:**
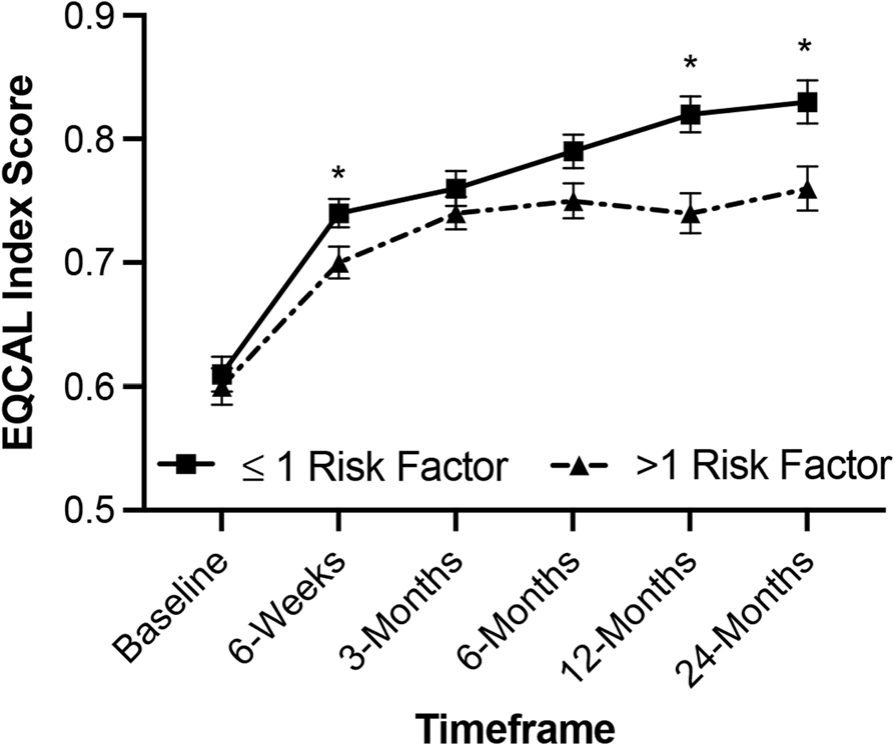
Improvement in EQ-5D by risk factor. Subjects in the low-risk (≤ 1 risk factors) and high-risk (> 1 risk factors) groups showed significant improvements in EQ-5D scores at all timepoints through Month 24 (*p < *0.0001). Improved scores were observed in the low-risk group at 6-weeks (*p =* 0.046), 12-months (*p =* 0.0005) and 24-months (*p =* 0.002) compared to the high-risk group. * *p < *0.05, between group comparison

Mean pre-operative EQ-5D scores in the low-risk group was 0.61 ± 0.16 and improved to 0.83 ± 0.17 (*p < *0.0001) at 24 months. Mean pre-operative EQ-5D scores in the high-risk group was 0.60 ± 0.17 and improved to 0.76 ± 0.18 (*p < *0.0001) at 24 months. Subjects in both groups (low and high-risk) showed significant improvements in EQ-5D scores at all timepoints (*p < *0.001). There was no statistical difference in EQ-5D outcomes between risk factor groups at baseline (*p > *0.05). Improved EQ-5D scores were observed in the low-risk group at 6-weeks (*p* = 0.046), 12-months (*p =* 0.0005) and 24-months (*p =* 0.002) compared to the high-risk group (Fig. 1).

#### Disability score (ODI)

**Fig. 2:**
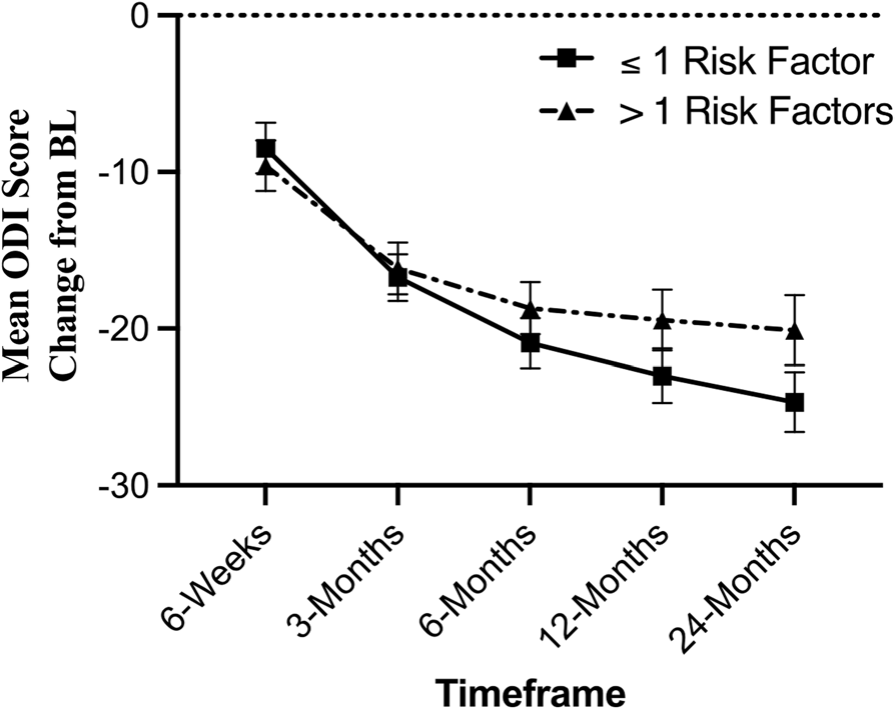
Improvement in ODI by risk factor. Subjects in the low-risk (≤ 1 risk factors) and high-risk (> 1 risk factors) groups showed significant improvements in ODI scores at all timepoints extending to Month 24 (*p < *0.0001). A between group comparison that accounted for baseline differences between groups showed no significant changes at any timepoint between groups, *p > *0.05

A statistical difference (*p =* 0.02) in ODI at baseline was observed between the low-risk group (63.8 ± 16.4) and high-risk group (67.9 ± 16.8), *p =* 0.02. Mean pre-operative ODI scores in the low-risk group improved to 37.6 ± 18.5 (*p < *0.0001) at 24 months. Mean pre-operative ODI scores in the high-risk group improved to 47.9 ± 20.5 (*p < *0.0001) at 24 months. Subjects in both groups (low and high-risk) showed significant improvements in ODI scores at all timepoints (*p < *0.0001). Analysis which adjusted for baseline group differences looked at change from baseline comparisons between groups showed no significant differences between low-risk and high-risk groups (Fig. 2).

#### VAS leg and back pain

Mean pre-operative VAS-Back pain scores in the low-risk group was 57.3 ± 27.5 and improved to 11.5 ± 20.8 (*p < *0.0001) at 24 months. Mean pre-operative VAS-Back scores in the high-risk group was 57.6 ± 12.5 and improved to 12.5 ± 20.8 (*p < *0.0001) at 24 months. Subjects in both groups (low and high-risk) showed significant improvements in VAS-back scores at all timepoints extending to month 24 (*p < *0.0001). There was no statistical difference in VAS-Back pain score outcomes between risk factor groups at baseline (*p > *0.05). A significant difference was noted between risk groups at 24 months (*p =* 0.04) with the low-risk group showing further reduction in back pain scores compared to the high-risk group.

**Fig. 3:**
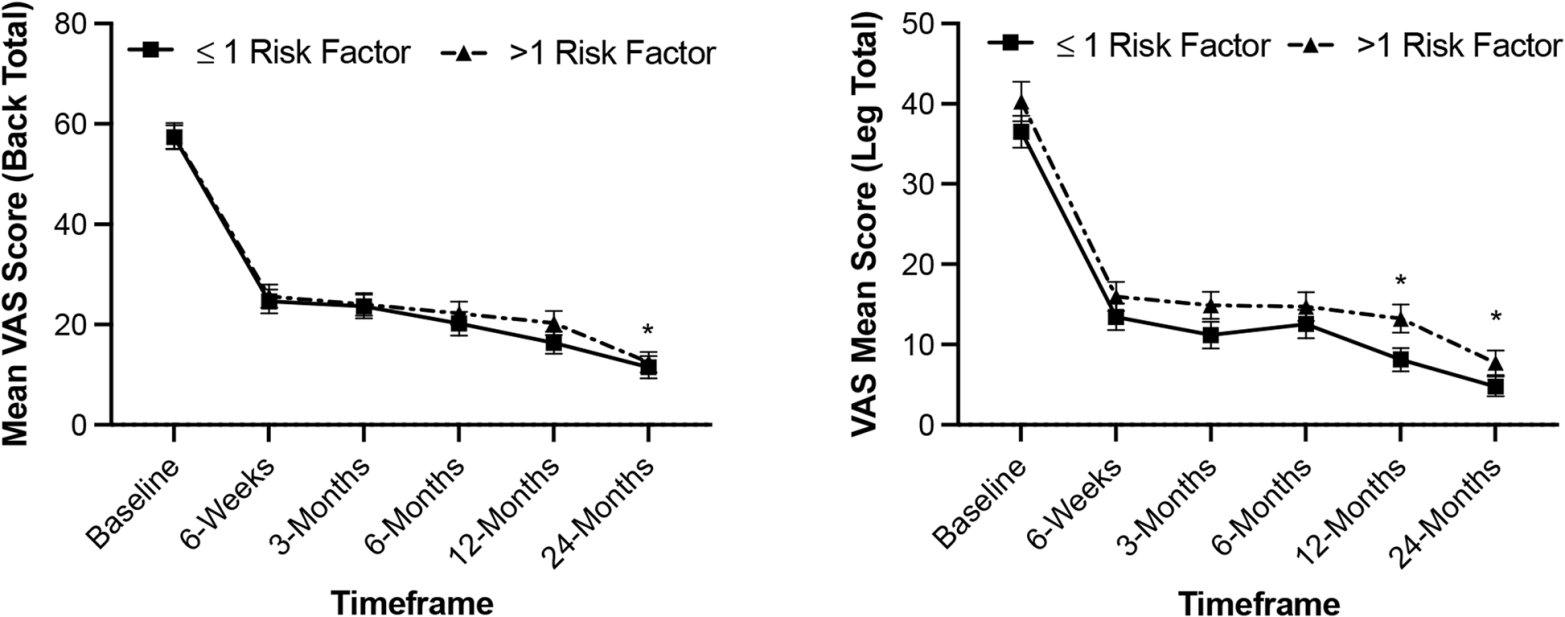
Improvement in VAS by risk factor. Subjects in the low-risk and high-risk groups showed significant improvements in VAS pain scores for Back and Leg (Total) at all timepoints extending to Month 24 (*p < *0.0001). Improved scores were observed in the low-risk group in VAS-Back pain at 24-months (*p =* 0.04) and in VAS-Leg pain at 12-months (*p =* 0.003) and 24-months (*p =* 0.03) compared to the high-risk group. * *p* < 0.05, between group comparison

Mean pre-operative VAS-leg pain scores in the low-risk group was 36.5 ± 23.0 and improved to 4.8 ± 12.0 (*p < *0.0001) at 24 months. Mean pre-operative VAS-leg scores in the high-risk group was 40.3 ± 36.5 and improved to 7.7 ± 15.6 (*p < *0.0001) at 24 months. Subjects across all groups (low and high-risk) showed significant improvements in VAS-leg scores at all timepoints extending to Month 24 (*p < *0.0001). A significant difference was noted between groups at 12 months (*p =* 0.003) and 24-months (*p =* 0.045) with the low-risk group showing further reduction in leg pain scores compared to high-risk subjects (Fig. 3).

#### Safety outcomes

A total of 665 AEs were reported through the 24-month follow-up period. The most common AE reported was pain (53 events, 8%) and back pain (37 events, 5.6%). No other category of adverse events exceeded 2%. AEs were generally considered common for this patient population. The occurrence of AEs among subjects were similar among risk factor groups (high-risk, *n* = 131, 93.6%; low-risk, *n* = 132, 98.5%). The majority of subjects did not have an AE related to the bone graft (*n* = 263, 96.0%). A total of 9 subjects had an AE that was related to the bone graft with a higher percentage observed in the high-risk group (high-risk, *n* = 7, 5.0%; low-risk, *n* = 2, 1.5%). A mean of 2.31 (SD 3.4) AEs were reported in the high-risk group and 1.88 (SD 3.2) AEs in the low-risk group. The total number of AEs experienced by subjects was not statistically different among risk groups (*p =* 0.15). A limited number of serious adverse events (SAE) considered related to the bone graft were observed (*n* = 2, 0.7%), both in the high-risk group. The SAE considered probably related to the bone graft occurred at 24-months and was listed as a second surgery. The SAE considered definitely related to the bone graft was worsening radiculopathy with the onset at the 6-week visit. Surgical exploration revealed that the bone graft migrated from the disc space. The subject was revised and went on to successful fusion. No statistically significant interaction between the development of a related SAE and number of risk factors was observed (*p =* 0.10) (Table 4).

**Table 4:** Adverse events by risk factor. Similar adverse event reporting was noted between risk factor groups

Variable	> 1 Risk Factors *n* = 140	≤ 1 Risk Factor *n* = 134	Overall *n* = 274	*p*—value
**Adverse Events**				
Not Related to Bone Graft	131 (93.6)	132 (98.5)	263 (96.0)	0.10
Related, Not Serious	7 (5.0)	2 (1.5)	9 (3.3)	
Related, Serious	2 (1.4)	0 (0.0)	2 (0.7)	
**Number of Adverse Events by Patient**				
Mean (SD)	2.31 (3.3)	1.88 (3.2)	2.10 (3.3)	0.15
Min–Max	0–21	0–22	0–22	

## Discussion

Advances in medicine have allowed more extensive and complex procedures to be performed in at-risk populations including procedures for lumbar spinal surgery. Lumbar spinal procedures can be complicated with a myriad of adverse effects associated with the procedure and recovery. The identification of predictors of complications, poor outcomes, and non-union requires risk assessment for implementing appropriate preventative measures in at-risk populations and best operational procedures. The development of new modifiable factors that may improve surgical and patient outcomes and achieve comparable results to patients that are low-risk are of interest. The selection of CBA as a primary bone graft choice for patients undergoing spinal surgery with known risk factors poses to provide many advantages. The current study explored the impact of CBA for lumbar spinal fusion as assessed by dynamic radiographs and CT and effect on clinical outcomes in patients stratified by risk factor.

Various clinical and demographic risk factors impact on the rate of non-union. Risk factors for complications and their association with age were analyzed in a retrospective analysis of patients who underwent lumbar spinal fusion surgery and found a complication rate of 15.0%. The rate of operative complications was significantly higher in patients 70 years of age or older than in other age groups [19]. A meta-analysis review of sixteen studies involving 13,393 patients shows that obesity and BMI were independent risk factors for complications including surgical site infections in patients who had undergone lumbar spine surgery. A retrospective review conducted by Wang et al. evaluated the rates and indications of reoperations following primary lumbar fusion in addition to independent risk factors for early and late reoperation. Multivariable analysis showed that osteoporosis was independently associated with early reoperation and that multilevel fusion was independently associated with late reoperation [20]. A review of clinical studies that present fusion rates following at least one-year follow up reported non-union as the most frequently reported complication at 14.0% (successful fusion of 86.0%) [21]. Patients undergoing spinal surgery who present with risk factors for non-union are more likely to show a reduced fusion rate or increased complication profile peri- and post-operatively [22–24]. Fusion rates at one-year post-operation in current smokers show reduced rates of fusion compared to non-smokers [25–27]. Brown et al. report a significant reduction in fusion rates at 1–2 years post-operation in smokers (40%) compared to non-smokers (8%) [25]. Diabetic patients show increased complications with multilevel fusion and significantly greater non-union rates (22–26% non-union) compared with non-diabetic patients (5% non-union) [28]. Indeed, it has been reported that with multilevel fusion, each additional level of fusion required decreases successful spinal fusion by ~ 20% [29]. The current study found a successful fusion rate of 90.0–93.9% among low-risk and high-risk groups following surgical procedures using CBA as the primary (> 50% by volume) bone graft substance. Of the risk factors analyzed (i.e., nicotine use, diabetes, osteoporosis, multiple levels (surgery), multiple risk factors, BMI, and age), no risk factor was significantly associated with a difference in the number of subjects that fused versus those that had failed fusion in either group. Therefore, regardless of risk factor, surgery using CBA resulted in comparable rates of fusion among low and high-risk groups. Risk factors for non-fusion did not result in lower fusion success.

Improvements in patient-reported clinical outcomes included quality of life, well-being and pain scores. Clinical outcome improvement was observed in both risk groups regardless of the number of risk factors reported. Greater improvements were observed in the low-risk group when compared to the high-risk group in pain scores. Subjects with multiple risk factors may not perform as well as subjects with a low number or no risk factors, however our findings showed significant improvements regardless of associated risk. The low-risk group showed a greater improvement compared to the high-risk group at several timepoints in all clinical outcomes assessed. These findings are in keeping with other reports showing that individuals with risk factors have a poorer clinical prognosis following surgery [22–25].

Risk factor impact on spinal surgery outcomes following the use of CBA exclusively has not been explored. Autograft substitutes provide all the key grafting elements necessary for bone repair. CBA is derived from donors that must pass stringent screening criteria. In contrast, allograft from at-risk patients may be compromised in quality. Further exploration is necessary to elucidate the mechanisms at play that may provide better outcomes for at-risk patients using CBA for lumbar spinal surgery.

Altogether, a limited number of serious adverse events considered related to the bone graft were observed, both in the high-risk group. The number of adverse events were similar among risk factor groups. A limited number of serious adverse events were observed which exclusively were seen in the high-risk group. These AEs were common and in keeping with this patient population.

The current study design employed the use of CBA as the primary (> 50% by volume) bone graft substance for surgery. While study outcomes aimed to investigate the overall efficacy of CBA on fusion rates and patient-reported outcomes stratified by patient groups with risk factors, the lack of a control arm limits direct comparisons to other graft materials and gross interpretation of the data. Direct comparisons between CBA and other bone graft materials are not supported by this design and was not the goal of the study. Therefore, it is difficult to attribute the success of fusion or satisfaction of patients to the applied allograft. While the choice of bone graft is an important consideration in surgical procedures, patient outcomes are not solely dependent on the bone graft. Numerous factors contribute to patient success including the surgical technique employed, risk factors and post-operative care. The multifaceted nature of surgical success provides challenges to distinguish individual contributions to success, each contributor is not independent of one another. Results must consider these other factors that may contribute to positive findings. In addition, there is likely an independent impact of the graft vs. risk factors on patient outcomes. Further studies are warranted to determine superiority of efficacy in these regards.

## Conclusions

Fusion success and subsequent patient benefits following lumbar spinal surgery can be significantly impacted by certain risk factors. Risk factors place patients in danger of non-union, surgical complications, and reduced post-surgical patient success. Identification and investigation into risk factors and new surgical initiatives to mitigate these effects pose to be of interest with high impact in the field. The current study provides additional support for the viability and efficacy of CBA in spinal fusion with new evidence highlighting benefit in subjects at-risk. Surgical intervention using CBA resulted in high rates of fusion, patient-reported outcomes, and safety measures regardless of risk factors present. CBA presents a viable graft modality that is not impacted by various risk factors. These data further support evidence of high rates of fusion success using CBA and promotes further exploration into this alternate modality as a beneficial source for bone graft in lumbar spine fusion with positive effect in high-risk patients.

## Data Availability

The datasets used and/or analyzed during the current study are available from the corresponding author on reasonable request.
